# Collapsing Aged Culture of the Cyanobacterium *Synechococcus elongatus* Produces Compound(s) Toxic to Photosynthetic Organisms

**DOI:** 10.1371/journal.pone.0100747

**Published:** 2014-06-24

**Authors:** Assaf Cohen, Eleonora Sendersky, Shmuel Carmeli, Rakefet Schwarz

**Affiliations:** 1 The Mina & Everard Goodman Faculty of Life Sciences, Bar-Ilan University, Ramat-Gan, Israel; 2 Department of Chemistry, Tel-Aviv University, Israel; Mount Allison University, Canada

## Abstract

Phytoplankton mortality allows effective nutrient cycling, and thus plays a pivotal role in driving biogeochemical cycles. A growing body of literature demonstrates the involvement of regulated death programs in the abrupt collapse of phytoplankton populations, and particularly implicates processes that exhibit characteristics of metazoan programmed cell death. Here, we report that the cell-free, extracellular fluid (conditioned medium) of a collapsing aged culture of the cyanobacterium *Synechococcus elongatus* is toxic to exponentially growing cells of this cyanobacterium, as well as to a large variety of photosynthetic organisms, but not to eubacteria. The toxic effect, which is light-dependent, involves oxidative stress, as suggested by damage alleviation by antioxidants, and the very high sensitivity of a catalase-mutant to the conditioned medium. At relatively high cell densities, *S. elongatus* cells survived the deleterious effect of conditioned medium in a process that required *de novo* protein synthesis. Application of conditioned medium from a collapsing culture caused severe pigment bleaching not only in *S. elongatus* cells, but also resulted in bleaching of pigments in a cell free extract. The latter observation indicates that the elicited damage is a direct effect that does not require an intact cell, and therefore, is mechanistically different from the metazoan-like programmed cell death described for phytoplankton. We suggest that *S. elongatus* in aged cultures are triggered to produce a toxic compound, and thus, this process may be envisaged as a novel regulated death program.

## Introduction

Phytoplankton mortality serves an important role in driving biogeochemical cycles in aquatic ecosystems. The process of phytoplankton blooming and sequential rapid collapse, allows nutrient recycling and very high productivity relative to the biomass [1]–[5]. A growing body of evidence implicates various factors in the abrupt termination of phytoplankton blooms, including abiotic stressors (e.g. nutrient limitation or intense light), and biotic factors (e.g. grazers and viruses). Additionally, algicidal bacteria also play an important role in phytoplankton mortality [6], [7].

In particular cases, cell death associated with culture demise, exhibits characteristics of metazoan programmed cell death (PCD). For example, proteolytic activity of 'caspases', which play a central role in metazoan PCD, was reported in cultures of the green algae, *Dunaliella tertiolecta* [8], *Dunaliella viridis* [9] and *Chlamydomonas reinhardtii* [10], [11], in the dinoflagellate, *Peridinium gatunense* [12], and in the cyanobacterium, *Trichodesmium sp.* [13]. A recent study reported a broad spectrum of caspase homologs (metacaspases) in phytoplankton and suggests diverse origin of these cell death proteases [14]. An excreted thiol protease was shown to be involved in the programmed death process of *P. gatunense* [15]. In addition, a cell suicide response was reported in *Anabaena flos-aquae* upon salt stress [16], in *Synechococcus* sp. strain PCC 7002 grown in the presence of urea [17], and in *Synechocystis* PCC 6803 under heat stress [18].

The role of viruses as a major cause of phytoplankton mortality has been recognized in recent years [2], [19], [20]. Interestingly, interaction between autocatalytic PCD and lytic viral infection was demonstrated in the coccolithophorid, *Emiliania huxleyi*. PCD in these cells occurs in response to the accumulation of virus-derived glycosphingolipids and recruitment of cellular metacaspases [21]–[23]. In the case of the cyanobacterium *Microcystis aeruginosa*, cyclic peptides provoke lysis via induction of virus-like particles [24].

The issue of phytoplankton loss in freshwater systems received much attention about three decades ago, due to the difficulty in explaining phytoplankton biomass based only on growth, grazing and sinking [25]. Relatively little is known, however, regarding the regulation of cell death in native populations and laboratory cultures of these species. Here, we describe different fates of aged cultures of the freshwater unicellular cyanobacterium *Synechococcus elongatus*: aging cultures either rapidly collapsed or gradually lost pigmentation and survived for longer periods. We demonstrate that the extracellular fluid of moribund cells (conditioned medium, CM) has a toxic effect on exponentially growing cultures of *S. elongatus* as well as on various phytoplankton species. The extracellular toxic compound(s) are thus implicated in mediation of a regulated death process.

## Results

### Light-dependent toxic effect of CM from collapsing culture

**Figure 1:**
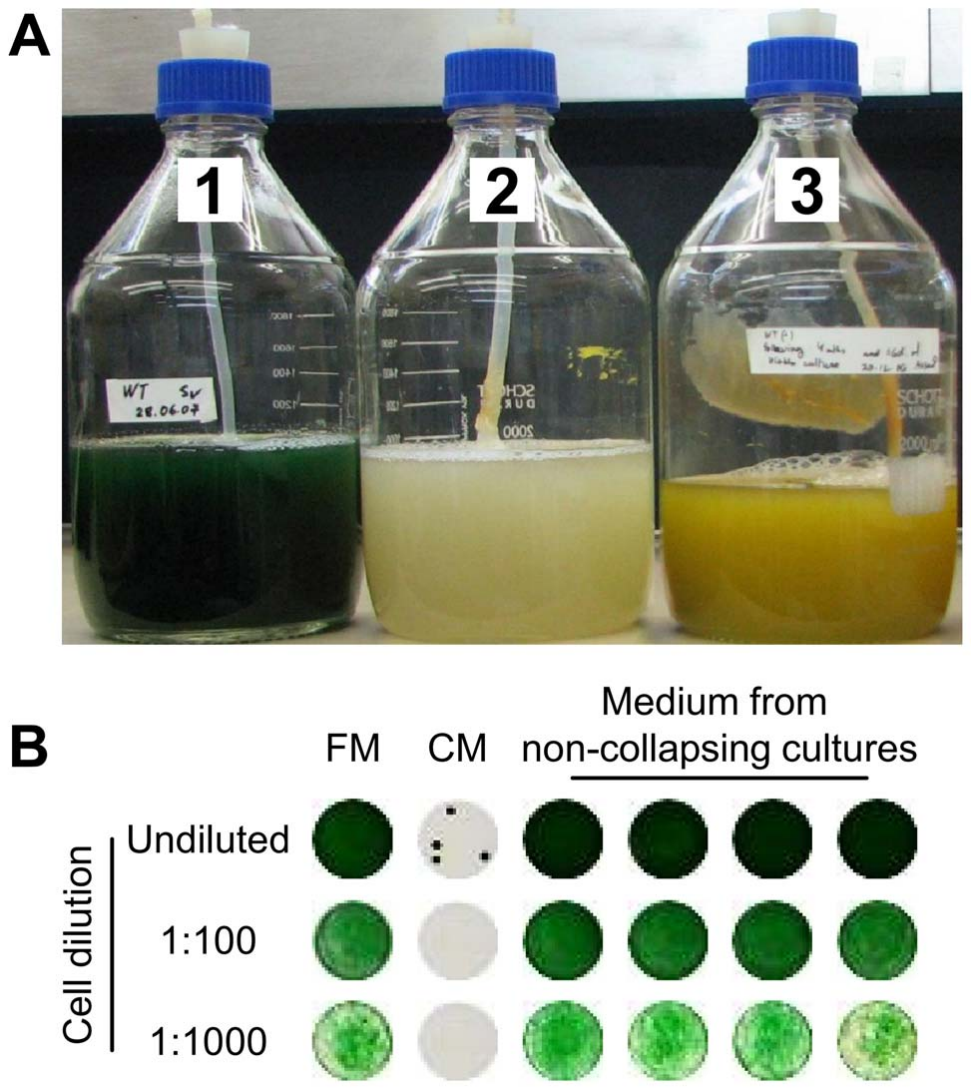
Different fates of aged cultures of the cyanobacterium *S. elongatus*. (**A**) Cultures maintained at stationary phase were characterized by dark blue-green pigmentation up to about 3 months (culture No. 1). Older cultures either rapidly collapsed (culture No. 2), or gradually acquired a yellowish color (culture No. 3) and further survived. Cultures No. 1 and 2 are about 3 months old, whereas culture No. 3 is 6 months old. (**B**) Conditioned medium (CM) of a collapsing culture induced rapid cell death of exponentially growing cells of *S. elongatus* in contrast to medium from non-collapsing cultures, which did not affect viability. Cells were inoculated into fresh medium (FM) as a control. 5 µl of undiluted cultures or cultures diluted 1∶100 or 1∶1000 were 'spotted' on solid growth medium, following exposure to the different media (see Methods).

In our studies of *S. elongatus,* we noted that cultures maintained for about 3 months, exhibited one of the following fates. Some cultures changed within 3–4 days from dark blue-green to a whitish appearance (Fig. 1A, cultures No.1 versus 2, respectively), while others exhibited a gradual pigmentation change to a yellow-green color (Fig. 1A, culture No. 3) and survived for an additional 4–5 months. Collapsing cultures were clearly distinct from 'non-collapsing cultures' since by the time demise of a culture resulted in a whitish appearance, a 'non-collapsing culture' of the same age was still dark-green in color (note that culture No. 3 in Fig. 1A, which exhibits substantial pigmentation change, is 6 months old).

**Figure 2:**
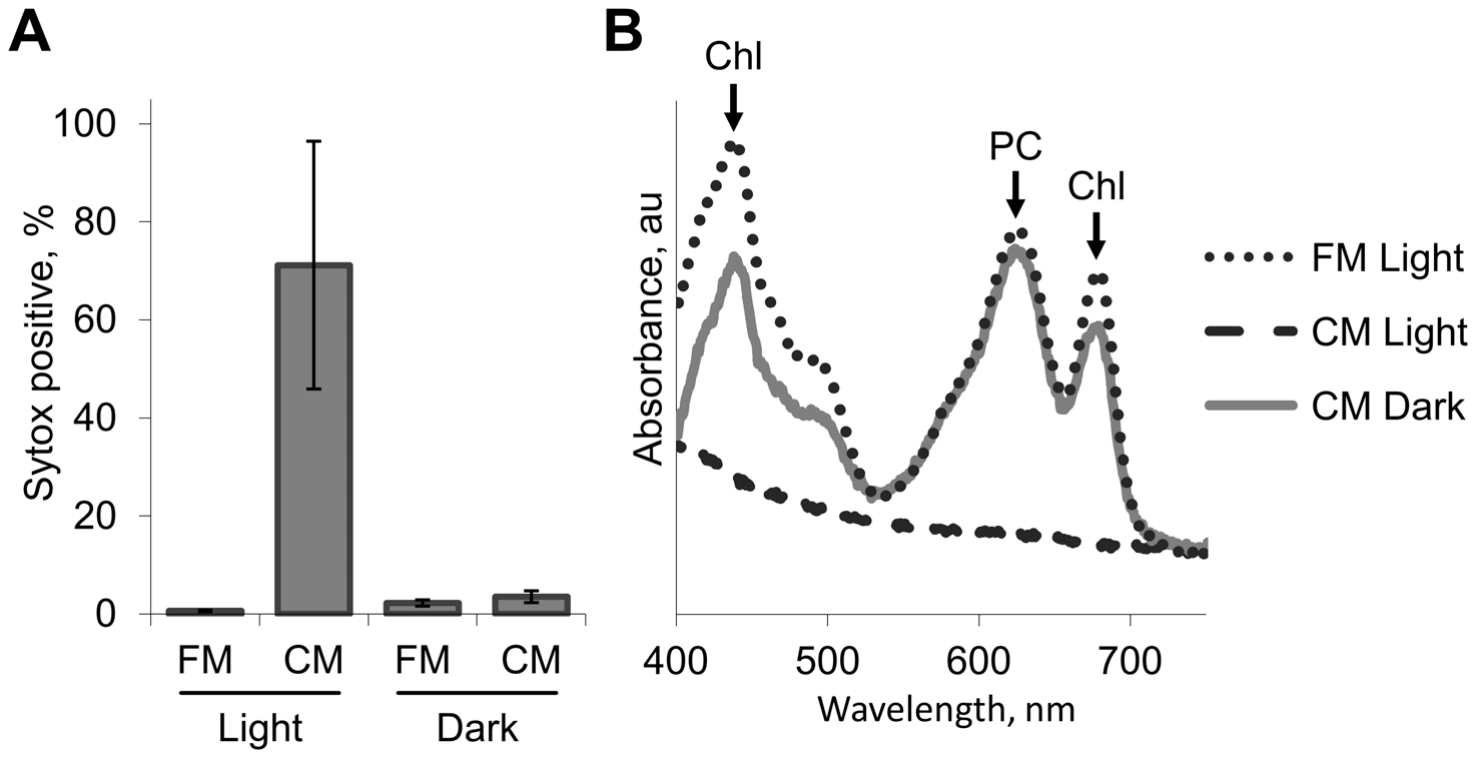
Light-dependent toxic effect of conditioned medium from collapsing culture. Viability assessment using Sytox green (**A**), and absorbance spectra (**B**) of exponentially growing cells 24 h after inoculation into fresh growth medium (FM), or into conditioned medium of a collapsing culture (CM). Exposure to CM under light resulted in bleaching of cell pigments including chlorophyll (Chl) and phycocyanin (PC).

The fast demise, which was exhibited by roughly half of the *S. elongatus* cultures, raised the hypothesis that cell death is propagated through the culture by compound(s) that are produced by the dying cells. Indeed, exposure of exponentially growing cells of *S. elongatus* to CM of a collapsing culture imposed rapid cell deterioration as observed by plating (Fig. 1B). Additionally, viability assessment using Sytox indicated compromised membranes of cells exposed to CM (Fig. 2A, light treatment). Intriguingly, the damaging effect of CM was light-dependent; exposure of cells to CM in the dark did not result in significant increase in Sytox positive cells, compared to cells inoculated into fresh medium (Fig. 2A, dark treatment). Additionally, the destructive effect of CM under illumination was apparent as complete de-pigmentation, as evident by visible absorbance spectra, whereas cell pigmentation was largely unaffected by incubation with CM in the dark (Fig. 2B).

The extracellular fluids collected from aging non-collapsing cultures (*e.g.* culture No. 3, Fig. 1A) were not harmful to exponentially growing cells (Fig. 1B).

### CM is harmful to large variety of photosynthetic microorganisms but not to eubacteria

**Figure 3:**
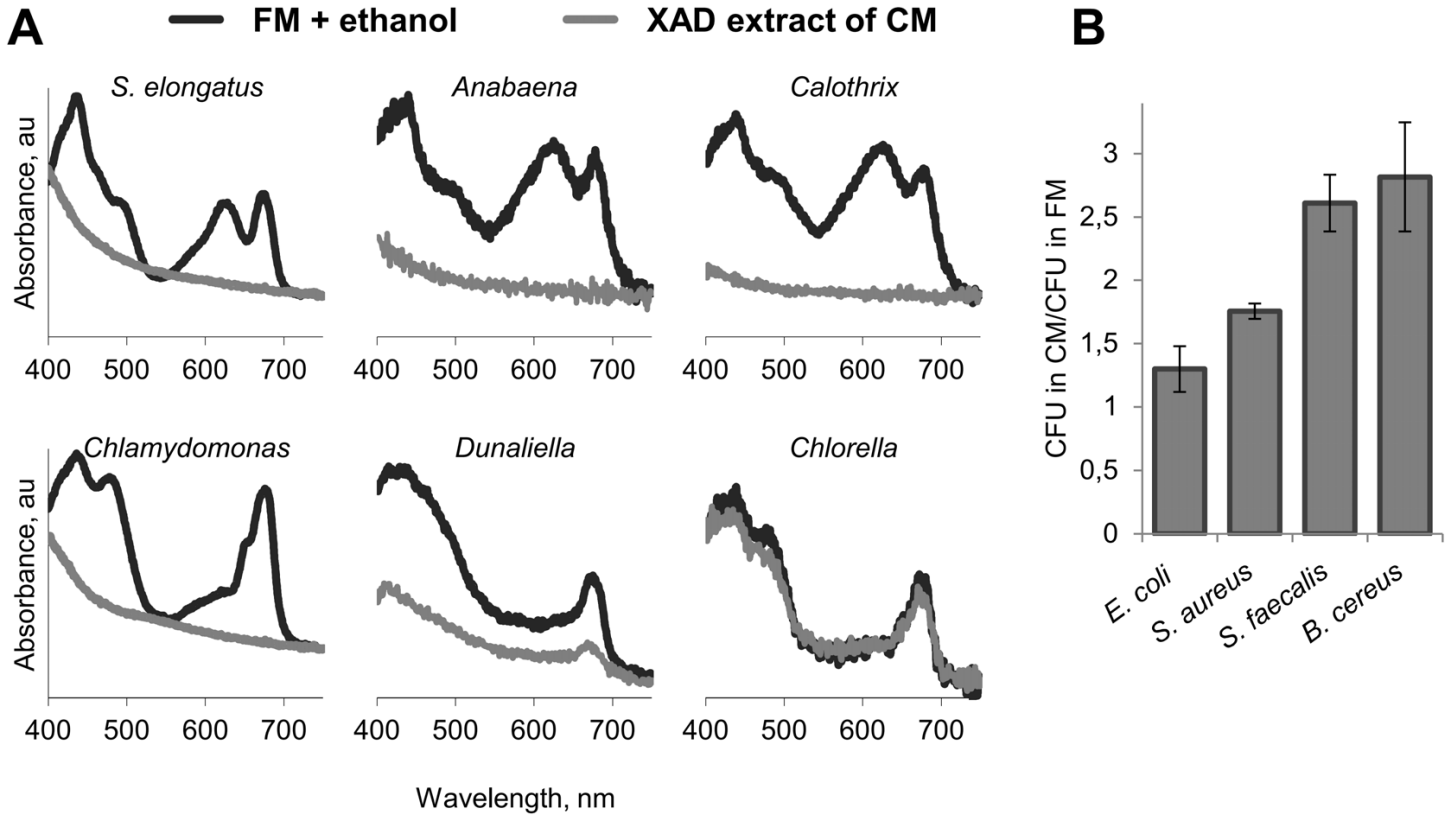
CM is harmful to photosynthetic microorganisms but not to heterotrophic bacteria. (**A**) XAD-extract of CM was applied to variety of cyanobacteria and algae including *S. elongatus, Anabaena* PCC 7120, *Calothrix* PCC 7601, *Chlamydomonas reinhardtii*, *Dunaliella salina* and *Chlorella vulgaris*. Sensitivity was examined spectroscopically to detect the effect on pigmentation. Additional phytoplankton species were examined, as well (see Table S1). Substances extracted with XAD were dissolved in ethanol; this organic solvent was added to fresh medium (FM) in the control samples. (**B**) *Escherichia coli*, *Staphylococcus aureus*, *Streptococcus faecalis* and *Bacillus cereus* were inoculated into FM or CM. The number of colony forming units (CFU) following 12 h illumination in CM was normalized to CFU obtained following exposure to FM.

The dependence of the toxic effect of CM on light raised the hypothesis that the photosynthetic electron transfer chain is involved in mediating the damage. We examined the effect of CM on a variety of phytoplankton species including cyanobacteria and algae, as well as on heterotrophic bacteria, to determine whether CM specifically affects photosynthetic organisms. All phytoplankton species examined, with the exception of *Chlorella vulgaris*, were sensitive to CM (Fig. 3A and Table S1). In contrast, the eubacteria examined were insensitive to CM, and in fact, CM supported some proliferation, likely due to organic material present in the extracellular milieu of the collapsing culture (Fig. 3B). Taken together, the sensitivity of phytoplankton (excluding *Chlorella*) and insensitivity of eubacteria to CM suggests that the toxic compound targets the photosynthetic electron transport chain (see Discussion).

**Figure 4:**
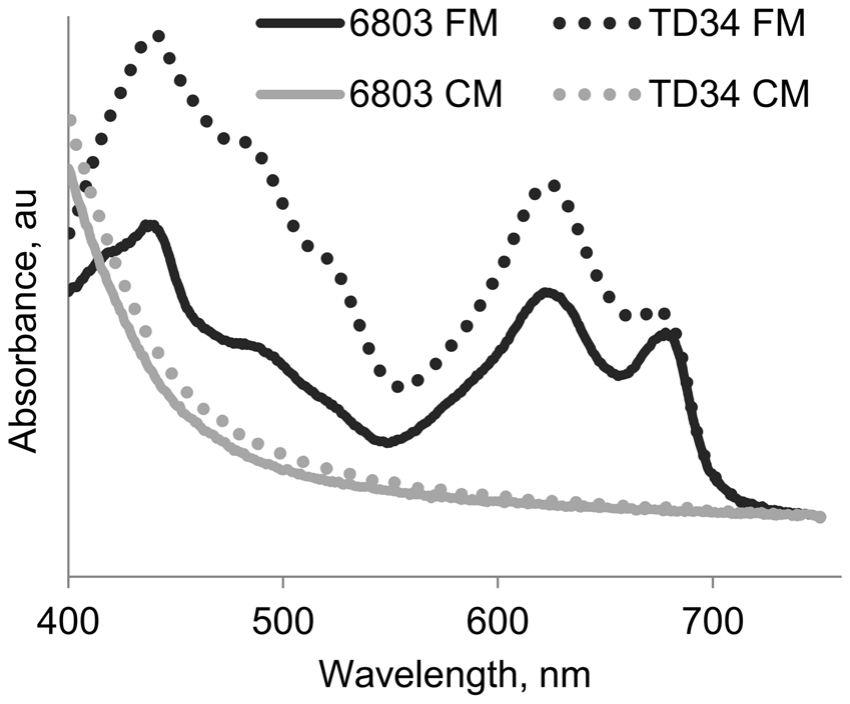
The TD34-mutant of *Synechocystis*, which lacks a functional photosystem II reaction center, remains sensitive to conditioned medium (CM). Shown are wild type *Synechocystis* PCC6803 (6803) and the mutant in which the three copies of *psbA*, each encoding the D1 protein of photosystem II, were inactivated (TD34). Cells were inoculated into fresh medium (FM) or CM.

The filamentous cyanobacterium *Scytonema hofmanni* produces a toxic substance, cyanobacterin, which is toxic to a variety of photosynthetic organisms [26], [27]. This substance was shown to act at the reaction center of photosystem II. To test whether the active compound in CM acts similarly, we examined the sensitivity of the TD34 mutant of *Synechocystis* PCC 6803, which lacks the essential protein of photosystem II, D1, and is grown heterotrophically [28], [29]. This mutant remained sensitive to CM (Fig. 4); thus, a functional photosystem II is not required to enable the damaging effect of CM.

### High density cultures survive CM

**Figure 5:**
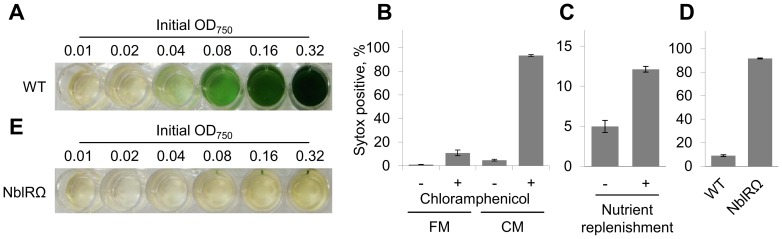
Cells respond to CM in a density dependent manner. (**A**) Exponentially growing cells of *S. elongatus* were inoculated into conditioned medium (CM) at different initial cell densities, as indicated by the optical density at 750 nm (OD_750_). (**B**) Chloramphenicol, an inhibitor of protein synthesis, impaired the ability of high density cultures to cope with the deleterious effect of CM. (**C**) Cells coped better with CM when it was not supplemented with nutrients. (**D and E**) Inactivation of the *nblR* gene, encoding a response regulator essential for nutrient starvation responses, results in extreme sensitivity to CM. Data shown in **B-D** represent cells exposed to CM at OD_750_ = 0.04. Note the different *y* axis scale in (**C**) versus (**B**) and (**D**).

Cell killing was observed only when cultures were inoculated into CM at a relatively low density (Fig 5A, cultures initiated at OD_750_ 0.01–0.02). At a higher cell density, however, cells survived (Fig 5A, cultures initiated at OD_750_ 0.04–0.32). Assessment of membrane permeability using Sytox indicated that at a relatively high cell density (OD_750_  =  0.04), only ∼5% of the cells were Sytox positive (Fig. 5B) compared to 50–100% in the case of exposure to CM at a low cell density (see Fig. 2A). Addition of the protein synthesis inhibitor, chloramphenicol, to a culture at a cell density that allowed survival following CM treatment, increased the fraction of Sytox positive cells to over 90% (Fig.5B). Thus, the ability of the cells to overcome the harmful effect of CM requires *de novo* protein synthesis. As noted above, very dense aged cultures abruptly collapsed (Fig. 1A, compare cultures 1 and 2); thus, we suggest that at this stage of culture growth, the cells are sensitive to the toxic compound despite high density.

We noted that upon prolonged incubation (∼5 d), at particular densities, the cultures turned yellowish in a response resembling acclimation to nutrient starvation, and involving degradation of the light harvesting antennae (Fig. S1, initial OD_750_ 0.04 and 0.08). The observed pigment degradation occurred despite nutrient repletion since conditioned medium was supplemented with all nutrients of the growth medium (see Materials and Methods). Based on this observation, we suggest that a substance present in the CM induces degradation of light harvesting pigments despite the nutrient replete state.

As indicated in the experiments described above, CM was regularly supplemented with nutrients; however, when cultures were not supplemented, we noted that the cells coped better with the toxic effect of CM (Fig. 5C). Based on these observations, we suggest that in the absence of added nutrients, acclimation responses beyond pigment degradation (which is also observed in nutrient replete CM, see Fig. S1), are elicited and provide the cells with mechanisms that enable them to overcome the damaging effect of CM. Support for this hypothesis is provided by the NblR-mutant, which is inactivated in a response regulator essential for nutrient starvation responses [30]; this mutant was extremely sensitive to CM, and did not survive at even very high cell densities (Fig.5D and 5E).

### Antioxidants attenuate the harmful effect of conditioned medium

**Figure 6:**
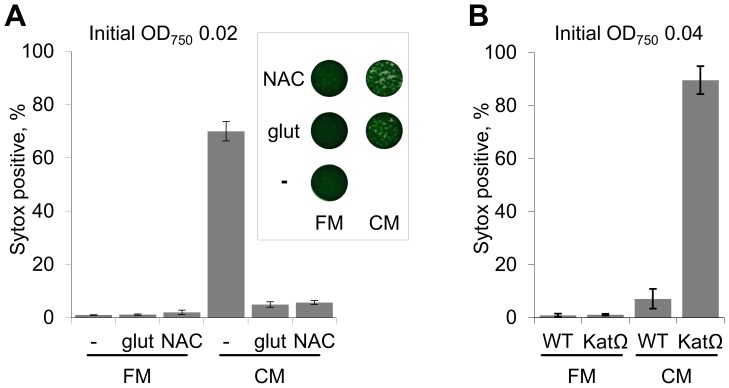
Antioxidants mitigate the toxic effect of CM. (**A**) The antioxidants glutathione (glut) and N-acetyl cysteine (NAC) alleviated the harmful effect of CM. Inset depicts assessment of viability by 'spotting' 5 µl of undiluted cultures onto fresh solid growth medium. (**B**) The catalase mutant strain, KatΩ, was highly sensitive to CM.

In light of the complete and rapid loss of all cellular pigments observed when low density cultures were exposed to CM (Fig. 2B), we raised the possibility that a reactive compound(s) produced by dying cells is responsible for destruction of the pigments and cell bleaching. To test involvement of oxidative stress, cultures were exposed to CM in the presence of the antioxidants glutathione or N-acetyl cysteine. These compounds mitigated the harmful effect of CM as revealed by Sytox staining (Fig. 6A) and plating on solid medium (Fig. 6A, inset). Based on this damage alleviation by antioxidants, we postulated that cellular antioxidative activity enables high-density cultures to cope with the deleterious effect of the CM. Indeed, a high-density culture of the KatG mutant, which lacks catalase-peroxidase activity [31], was highly sensitive to CM compared to the relative resistance of the wild type strain (Fig. 6B).

### Initial characterization of the toxic CM component and its mode of action

In an attempt to characterize the molecular nature of the active CM component, extraction of CM was performed using the polymeric adsorbent Amberlite XAD2, a resin that binds non-polar substances. This extract was cytotoxic as revealed by Sytox staining of treated cells (Fig. S2A). Furthermore, extraction of CM with chloroform indicated that the active compound equilibrated with the organic phase (Fig. S2B). Taken together, these analyses indicated the non-hydrophilic nature of the toxic substance. Additionally, the substance was heat resistant, as autoclave treatment of CM did not abolish the toxicity of CM (Fig. S2C). Attempts at purification of the active substance were performed (see Materials and Methods); however, we have not been able to fully purify or characterize this substance.

**Figure 7:**
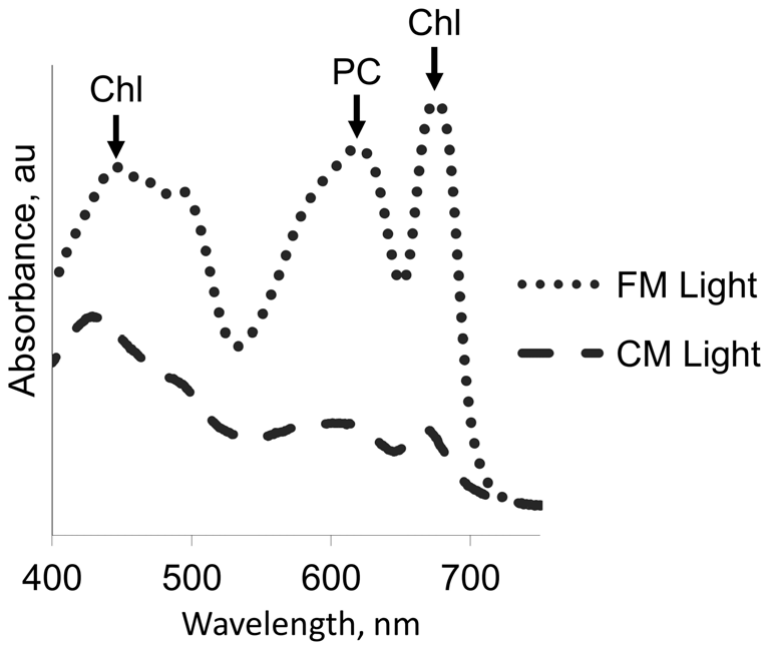
Conditioned medium causes bleaching of pigments in a cell extract. Absorbance maxima of chlorophyll (Chl) and phycocyanin (PC) are indicated.

In particular cases, bacterial or cyanobacterial cell death is mediated by the activation of a genetically encoded cell-death program. The destructive effect of conditioned medium, however, was also observed in a cell-free system, as revealed by pigment bleaching following exposure of a crude cell extract to CM (Fig. 7). These data clearly demonstrate that an intact cell is not required for the photobleaching effect of CM (see Discussion).

## Discussion

This study demonstrates that CM from collapsing cultures of *S. elongatus* has a cytotoxic effect on exponentially growing cells of this cyanobacterium, as well as on diverse cyanobacterial and algal species (Figs. 2 and 3A; Table S1). The toxic effect is light-dependent (Fig. 2), suggesting the involvement of the photosynthetic electron transport chain, consistent with the lack of sensitivity of eubacterial species examined (Fig. 3B). Furthermore, severe pigment bleaching occurred in response to CM in crude cell extract (Fig. 7). This experimental setting, in which carbon fixation reactions do not effectively occur, provides support for the suggestion that the damage is exerted through the photosynthetic electron transfer chain. Alleviation of cell damage by small antioxidants (Fig. 6A) and the increased sensitivity of a catalase-peroxidase mutant (Fig. 6B) support involvement of oxidative stress as an essential step in the cytotoxic process.

In addition, we demonstrate that *S. elongatus* cells may overcome the killing effect of CM when challenged at a relatively high cell density (Fig. 5). This cyanobacterium copes with H_2_O_2_ in a density-dependent manner [31]. Therefore, it is possible that high-density cultures effectively detoxify H_2_O_2_ produced in response to CM, while the KatG-mutant is sensitive even at high cell densities. Furthermore, chloramphenicol treatment renders high-density cultures sensitive to CM (Fig. 5B), indicating that *de novo* protein synthesis is required to equip the cells with the mechanisms involved in damage prevention or alleviation. The response regulator NblR is involved in regulation of at least some of the pivotal responses, as evident by the extremely high sensitivity of the NblR-mutant (Fig. 5D and 5E).

Bacterial cell death may occur either by a necrotic process, as a direct consequence of conditions that are incompatible with survival, *e.g.* chemical or physical insults, or in a regulated programmed response that is elicited under certain conditions [32]–[34]. The ability of *S. elongatus* to produce a lethal compound may be envisaged as a regulated death process; namely, cells do not deteriorate directly due to nutrient limitation in the aging culture. Rather, they are triggered to produce a substance that rapidly kills the cells. This regulated death process differs from a PCD mechanism requiring activation of gene expression, since changes reflecting the effect of CM on exponentially growing cells (Fig. 2) were also observed in a cell free system (Fig. 7). A scenario in which only some of the cells in an aged culture are triggered to produce a toxic compound, which propagates cell death throughout the culture, should not be excluded. It is yet to be determined whether the toxic compound is excreted, or released into the extracellular milieu upon lysis of the producing cells.

It is not known why some cultures collapse after approximately 3 months, while others survive (Fig. 1A). It is possible that some cultures are not triggered to produce the toxic compound, but rather, gradual pigment degradation occurs, providing amino acids and likely supporting survival of the nutrient limited aged culture. We suggest that pigment degradation rather than cell death is elicited by CM in exponentially growing cells exposed to CM at OD_750_ of 0.04 or 0.08 (Fig. S1). It is also possible that the surviving cultures represent CM-resistant mutants that effectively decompose the toxic compound.

The role of the described cell death process is as yet unknown, though several hypotheses may be suggested. It is possible that in open growth niches, the toxic compound is accumulated at lower concentration relative to the levels found in our batch cultures. Thus, some of the cells, probably those which are less fit, are eliminated, whereas those cells that are better equipped with survival responses and are not damaged (or are less impaired), persist. Therefore, as suggested earlier for programmed cell death in unicellular organisms, such a differential death process will have a population-level benefit [5], [32]–[34]. In addition, since other phytoplankton species are sensitive to CM of *S. elongatus*, the toxic compound may represent an allelopathic substance. At specific cell densities or in a particular physiological state, *S. elongatus* cells might survive the toxic effect better than competing species.

## Materials and Methods

### Culture conditions

For collection of CM, cultures of *Synechococcus elongatus* were grown as follows: 1L culture was set in a 2L Schott Duran glass bottle with a screw cap, in which a hole was drilled to fit a silicone stopper (see Fig. 1A). Two holes were drilled in the stopper – one for silicone tubing for bubbling 5% CO_2_ in air into the culture, and another for insertion of short silicone tubing to prevent pressure from building up in the culture headspace. Cotton wool was inserted into the upper part of the tubing. This 'bubbling set' was wrapped with aluminum foil and autoclaved, whereas the bottle with the growth medium was autoclaved separately with an intact screw cap. Upon culture inoculation in the sterile hood, a 0.22µ filter was attached to the silicone tubing for bubbling of the CO_2_ enriched air. Compressed air from an oil free air compressor (Assouline Compressors Ltd, Model vs 204 50) was mixed with CO_2_ (Gordon Gas Ltd.) to yield 5% CO_2_ in air using flowmeters (Mego Afek) and the mixed air was humidified prior to bubbling into the culture.

For assessment of the effect of CM, 40 ml cultures were grown as follows: *S. elongatus* and all mutants derived from this strain, *Synechocystis* PCC 6803, *Anabaena* PCC 7120 and *Calothrix* PCC7 7601 were grown in BG11 medium in Pyrex tubes under bubbling with 5% CO_2_ in air as described earlier [35]. For growth of the TD34 mutant of *Synechocystis*, glucose was added to the medium (20%). Other phytoplankton species were grown in flasks under shaking as follows: *Chlorella vulgaris* was grown in Bristol medium [36]. *Synechococcus* WH 8102, *Nannochloropsis* sp., *Dunaliella salina*, *Thalassiosira weissflogii* and *Naviculla lenzii* were grown in F2 medium. In the latter two cases, silicates were added [37]. *Chlamydomonas reinhardtii* was grown in TAP medium [38].

*Escherichia coli*, *Staphylococcus aureus*, *Streptococcus faecalis* and *Bacillus cereus* were grown in Luria-Bertani (LB) growth medium.

### Collection of CM and toxicity assay

The study was conducted over the course of 2½ years, during which CM from 15 collapsing cultures was collected and characterized. Spotting of 5 µl from a collapsing culture did not yield colonies while a non-collapsing culture of the same age resulted in 'a lawn'. Prior to harvesting of CM, 5 µl cultures were 'spotted' on LB plates to screen for contaminants. Conditioned medium was further analyzed only when this assay did not indicate microbial contaminations. Collapsing cell cultures were centrifuged at 30,000 g for 15 min, the supernatant was passed through a 0.22 µm filter and stored at −20°C. Routinely, the CM was supplemented with all nutrients of fresh growth medium, prior to inoculation of exponentially growing cells. An exceptional experiment, in which we examined the effect of CM that was not supplemented with nutrients, is shown in Fig. 5C. Exponentially growing cells of *S. elongatus* were exposed to CM supplemented with all nutrients as well as 4 mM NaHCO_3_. Cultures (2 ml) in 24-well plates were illuminated (50 µmol photons m^−^^2^⋅s^−^^1^) or kept in the dark at 30°C. Unless indicated differently in the figure legend, cells were inoculated at OD_750_ = 0.02 (corresponding to ∼8*10^6^ cells/ml) and analyses were performed following 24 h of exposure to CM. To examine the effect of CM on cells at different densities, exponentially growing cells were concentrated by centrifugation (10500 g, 10 min) and resuspended to OD_750_ = 0.32. Two fold dilutions (in CM) of this cell culture resulted in a series of cell densities (see Fig. 5A). Viability was assessed by 'spotting' 5 µl of the undiluted culture as well as serial dilutions onto fresh solid growth medium (see Fig. 1B). Additionally, Sytox Green dead cell stain (Molecular Probes), a dye that enters cells with compromised membranes and binds to nucleic acids, was employed for viability assessment. For Sytox staining, cells were diluted with phosphate saline buffer to OD_750_ ∼0.0004. Following addition of Sytox (40 nM), cells were incubated in the dark for 15 min and analyzed by flow cytometry using Becton Dickinson FACS Calibur (excitation 488 nm, emission 530±15 nm). Fluorescence was plotted *vs* side scattering; cells inoculated into fresh medium served to define 'Sytox negative cells' (Fig. S3, see legend). The effect of CM on pigmentation was determined by measuring absorbance spectra using a Cary 100 spectrophotometer equipped with an integrating sphere (Agilent Technologies). Where indicated, chloramphenicol (250 µg/ml), glutathione, or N-acetyl-cysteine (2 mM each) were added. Cell extracts for examination of the effect of CM in a cell-free system were prepared as previously described [39]. A sample of 70 µl extract was added to 2 ml of CM or fresh medium in a 24-well plate and incubated as described above.

For assessment of the effect of CM on eubacteria, over-night cultures were diluted 10 fold in Luria-Bertani growth medium and grown for 3 h with shaking. Next, samples from these cultures were added either into FM or into CM to yield OD_600_ = 0.02, and the diluted cultures were shaken under illumination at 30°C for 12 h. Samples from serial dilutions (up to 10^−7^) were plated. Depending on the particular bacterium, single colonies were counted from dilutions 10^−3^, 10^−4^ or 10^−5^ and the colony forming units (CFU) in CM were normalized to CFU in FM.

Bar graphs represent averages (±standard deviations) of triplicates within a single experiment. All experiments reported were performed using at least three independent biological repetitions (in terms of the test cells used for assessment of the effect of CM, as well as the batch culture yielding the CM).

### Extraction and initial fractionation of CM

To extract the CM, Amberlite XAD-2 (Supelco) was added (20 g/L of CM); this mixture was agitated at room temperature for 80 min and filtered using Miracloth (Calbiochem). The resin was washed twice with acetone (0.5 L/L for each wash step). Wash fluids were collected by filtration, the acetone was evaporated using a hot water bath and the precipitates were resuspended with ethanol (200 µL/L). For assessment of toxicity, the XAD-extract was diluted 200 fold into fresh growth medium. Separation of XAD extract by Sephadex LH-20 yielded several active fractions. Further separation by high-pressure liquid chromatography (C-18, elution with water-acetonitrile gradient), did not allow purification and identification of the active compound. For chloroform extraction of CM, an equal volume of this organic solvent was added, and the mixture was shaken for 12 h at room temperature. The mixture was centrifuged in a 50 ml tube (4°C, 3300 g, 20 min), which was subsequently chilled at −80°C for 15 min. The chloroform phase was separated from the aqueous phase, evaporated in a hot water bath and the precipitates were resuspended with dimethyl sulfoxide (DMSO) (in 1/30 of the original volume extracted).

## Supporting Information

Figure S1 **Exposure of cells to CM at particular densities resulted in pigmentation change.** Cultures were photographed following 5d exposure to CM.
(TIFF)

Figure S2 **The active substance is non-hydrophilic and heat resistant.** (**A**) An XAD-extract of conditioned medium (CM) was added to a culture inoculated into fresh medium (FM) and the effect was assessed by Sytox staining. (**B**) Chloroform extract of CM was added to a culture inoculated into FM and the toxic effect was revealed by cell plating. (**C**) The active compound is resistant to autoclave treatment. Substances extracted with XAD or chloroform were dissolved in ethanol and dimethyl sulfoxide (DMSO), respectively (see Materials and Methods); these organic solvents were added to fresh medium (FM) in the control samples.
(TIFF)

Figure S3 **Flow cytometric analysis of Sytox treated cells inoculated into fresh medium (FM) or conditioned medium (CM).** Excitation was provided at 488 nm and emission measured at 530±15 nm (FL1). Fluorescence *vs* side scattering (SSC) is shown in a density plot. The horizontal line depicts the threshold for defining Sytox positive cells.
(TIFF)

Table S1 **Sensitivity of diverse cyanobacterial and algal species to CM.** Bleaching upon exposure to CM is indicated by +. *Chlorella vulgaris* was the only insensitive phytoplankton species (also see Fig. 3).
(DOCX)
