# Supplementary figures and images for: Collapsing Aged Culture of the Cyanobacterium Synechococcus elongatus Produces Compound(s) Toxic to Photosynthetic Organisms
Source: PLoS One. 2014 Jun 24;9(6):e100747. doi: 10.1371/journal.pone.0100747 (PMC4069110; doi:10.1371/journal.pone.0100747)

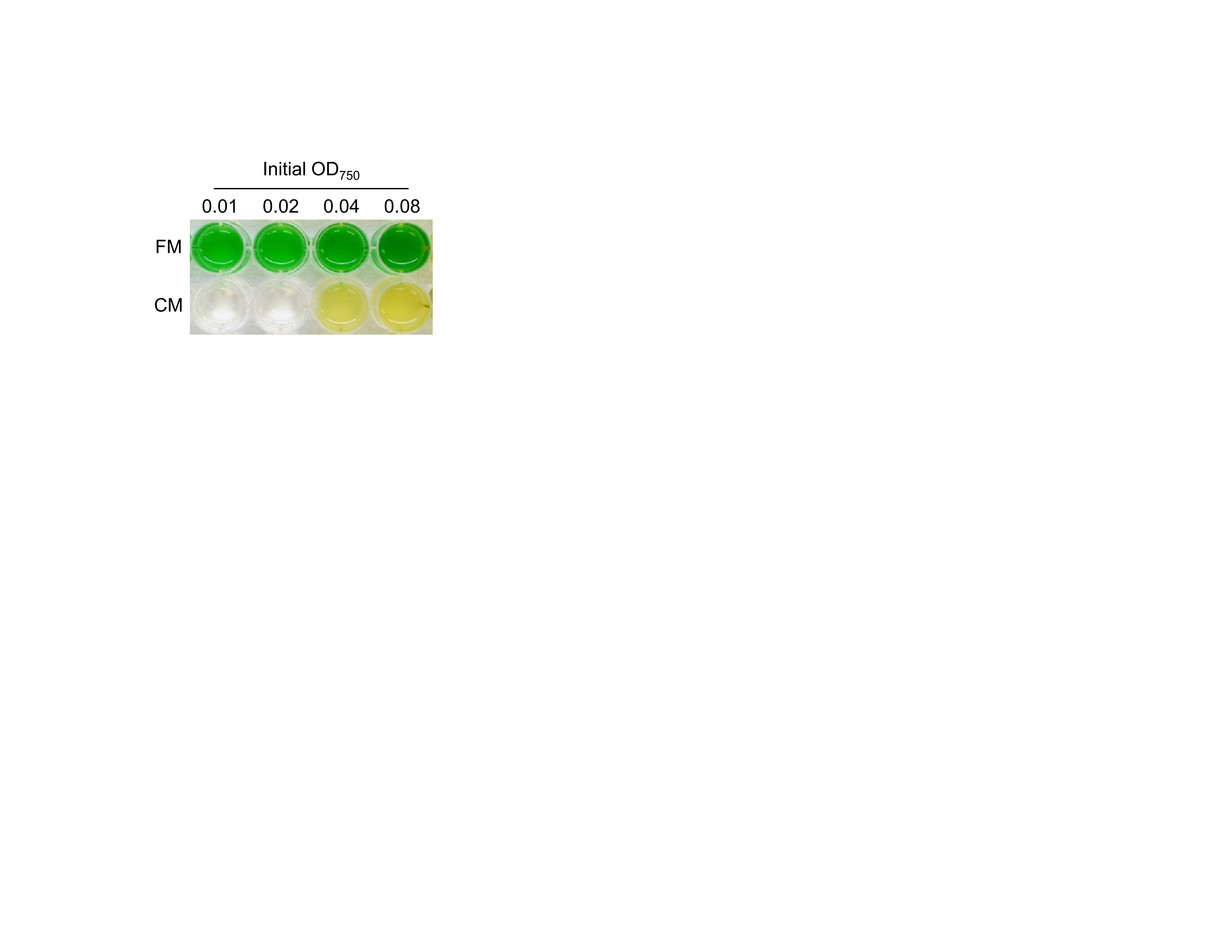

Supplement: Figure S1 — Exposure of cells to CM at particular densities resulted in pigmentation change. Cultures were photographed following 5d exposure to CM. (TIFF) [file pone.0100747.s001.tif]

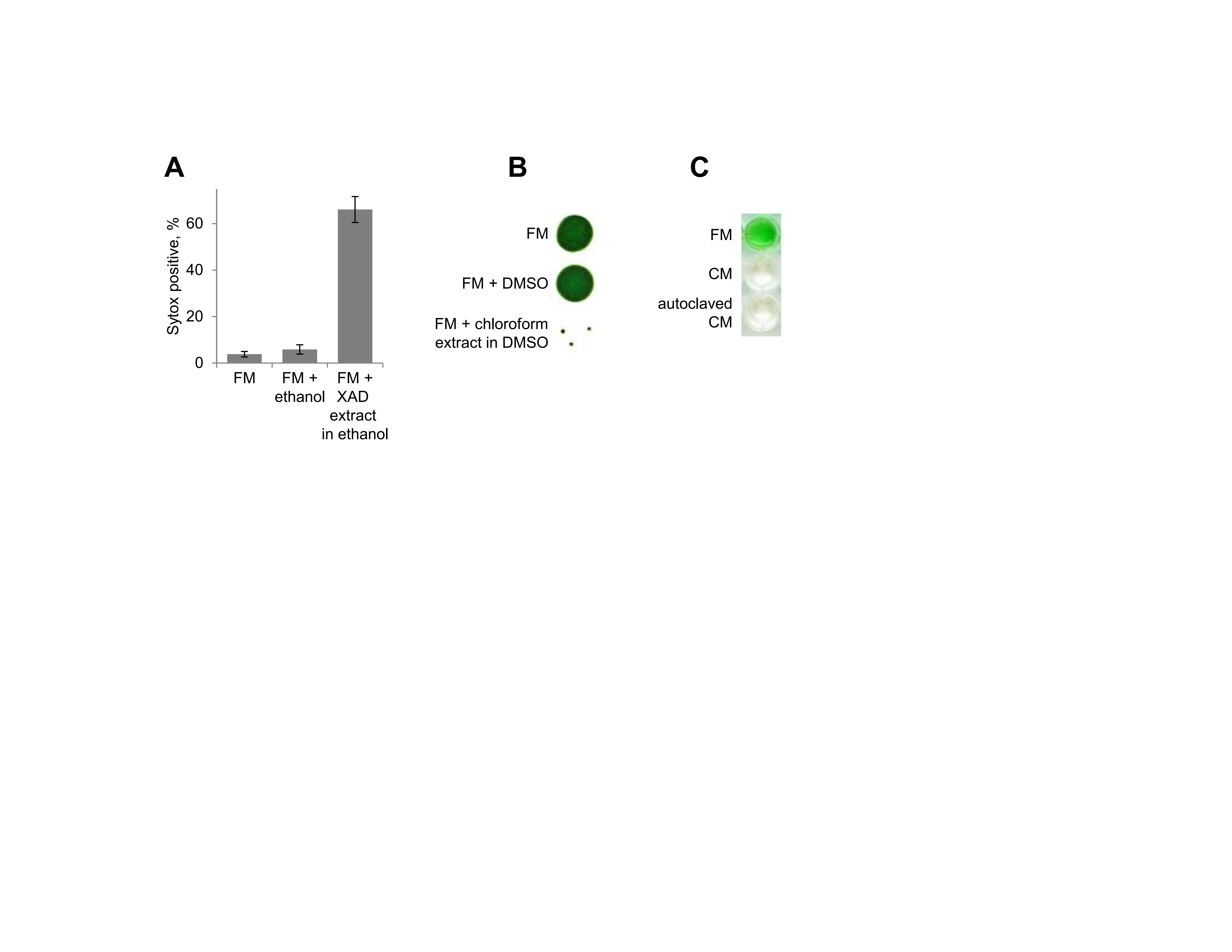

Supplement: Figure S2 — The active substance is non-hydrophilic and heat resistant. (A) An XAD-extract of conditioned medium (CM) was added to a culture inoculated into fresh medium (FM) and the effect was assessed by Sytox staining. (B) Chloroform extract of CM was added to a culture inoculated into FM and the toxic effect was revealed by cell plating. (C) The active compound is resistant to autoclave treatment. Substances extracted with XAD or chloroform were dissolved in ethanol and dimethyl sulfoxide (DMSO), respectively (see Materials and Methods); these organic solvents were added to fresh medium (FM) in the control samples. (TIFF) [file pone.0100747.s002.tif]

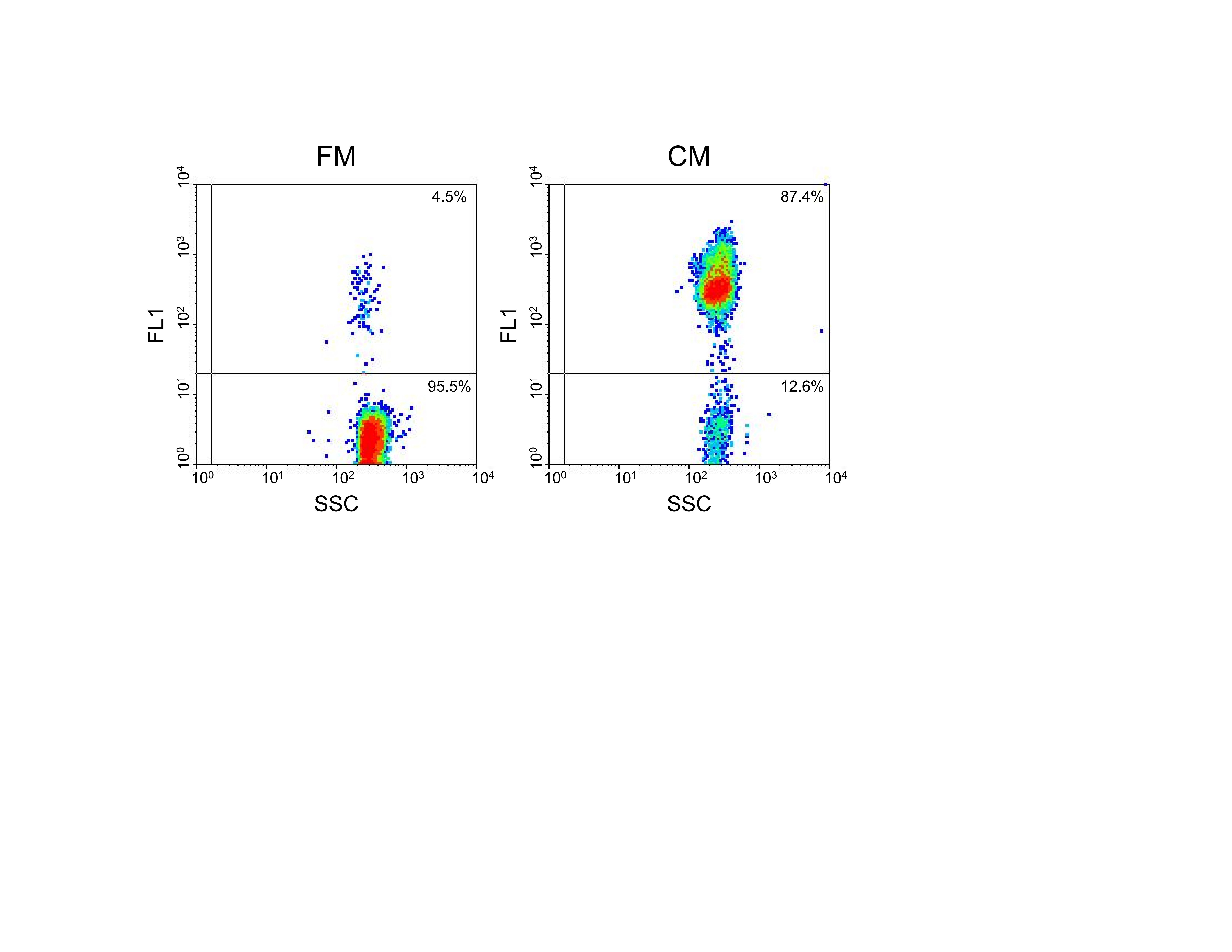

Supplement: Figure S3 — Flow cytometric analysis of Sytox treated cells inoculated into fresh medium (FM) or conditioned medium (CM). Excitation was provided at 488 nm and emission measured at 530±15 nm (FL1). Fluorescence vs side scattering (SSC) is shown in a density plot. The horizontal line depicts the threshold for defining Sytox positive cells. (TIFF) [file pone.0100747.s003.tif]
