# Supplementary material for: Collapsing Aged Culture of the Cyanobacterium Synechococcus elongatus Produces Compound(s) Toxic to Photosynthetic Organisms
Source: PLoS One. 2014 Jun 24;9(6):e100747. doi: 10.1371/journal.pone.0100747 (PMC4069110; doi:10.1371/journal.pone.0100747)
Supplement: Table S1 — Sensitivity of diverse cyanobacterial and algal species to CM. Bleaching upon exposure to CM is indicated by +. Chlorella vulgaris was the only insensitive phytoplankton species (also see Fig. 3). (DOCX) [file pone.0100747.s004.docx]

| Species examined | sensitivity |
| --- | --- |
| *Anabaena* PCC 7120 | **+** |
| *Calothrix* PCC 7601 | **+** |
| *Chlamydomonas reinhardtii* | **+** |
| *Chlorella vulgaris* | **-** |
| *Dunaliella salina* | **+** |
| *Nannochloropsis* sp. | **+** |
| *Naviculla lenzii* | **+** |
| *Synechococcus elongatus* PCC 7942 | **+** |
| *Synechococcus* WH 8102 | **+** |
| *Synechocystis* PCC 6803 | **+** |
| *Thalassiosira weissflogii* | **+** |

**Table S1: Sensitivity of diverse cyanobacterial and algal species to CM.** Bleaching upon exposure to CM is indicated by +. *Chlorella vulgaris* was the only insensitive phytoplankton species (also see Fig. 3).
